# Improving Pneumococcal Vaccination Rates among Rural Older Adults through Academic Detailing: Medicine, Nursing and Pharmacy Partnership

**DOI:** 10.3390/vaccines9040317

**Published:** 2021-03-31

**Authors:** Kimberly McKeirnan, Karen Colorafi, Zuan Sun, Kristopher Daratha, Darryl Potyk, John McCarthy

**Affiliations:** 1 College of Pharmacy and Pharmaceutical Sciences, Washington State University, Pullman, WA 99202, USA; 2 School of Nursing and Human Physiology, Gonzaga University, Spokane, WA 99202, USA; colorafi@gonzaga.edu; 3 School of Business, Whitworth University, Spokane, WA 99251, USA; zsun@whitworth.edu; 4 Daratha Analytics, Spokane, WA 99204, USA; daratha.analytics@gmail.com; 5 School of Medicine, University of Washington, Spokane, WA 99202, USA; potykd@uw.edu (D.P.); mccajf@uw.edu (J.M.)

**Keywords:** pneumococcal vaccination, rural healthcare, academic detailing

## Abstract

Academic detailing is an educational approach involving provision of evidence-based information by healthcare providers for healthcare providers with the goal of improving clinical decision-making. An interprofessional academic detailing initiative was developed to encourage rural providers to utilize guidelines when deciding which patients to vaccinate against pneumonia. This study utilized a quasi-experimental, single-group, pre-post observational design with physicians, nurses, and staff at two rural medical clinics. The 12-month academic detailing intervention included a needs assessment, workflow assessment of practice-based health information technology, vaccination training for providers and staff, and creation of exam-room posters encouraging patients to discuss vaccination with their provider. Six visits were made to deliver education, discuss needs, select priorities, and develop action plans from recommendations. Data were collected from each site for three years prior to the intervention year and for one year following the intervention. The annual rate of patients vaccinated increased during the five-year study. The cumulative proportion of the sample population that received vaccination also increased over time. Interprofessional academic detailing was well received and increased pneumococcal vaccination rates among rural-dwelling older adults. Given the alarming disparities in health outcomes for rural patients, educational outreach is needed to improve healthcare access and outcomes.

## 1. Introduction

Pneumonia is the leading cause of infection-related death in the United States and worldwide. Each year approximately one million American adults contract pneumonia, leading to 400,000 hospitalizations that carry an average cost of USD 10,962.50 per episode [1]. This year especially, pneumonia and respiratory illness are highlighted in discussion of the COVID-19 pandemic. The high mortality associated with COVID-19 is partly explained by bacterial pneumonia, which also caused the majority of deaths during the 1918 influenza pandemic [2]. Prior to the pandemic of 2020, death occurred in 5–7% of those who were hospitalized with pneumonia and mortality rates are even higher in patients age 65 years and older [3]. Healthy People 2020 set an aggressive goal to reduce the incidence of pneumococcal infections among older adults, targeting a goal of 31 cases per 100,000 people, a marked reduction from a 2008 observed baseline of 40.7 new cases per 100,000 adults. Achieving this goal requires increased vaccination against pneumococcal disease [4].

The National Foundation for Infectious Diseases (NFID) recognizes four key barriers to vaccinating adults with chronic conditions: competing priorities during patient visits, lack of ownership for necessary patient education, challenges in determining vaccination status, and complexity of recommendations [3]. While all are relevant to vaccination and worthy of discussion, the latter concern is particularly important with pneumococcal vaccination due to the multitude of factors a healthcare provider must consider before recommending vaccination (e.g., age, prior vaccination status, timing, and boosters). The complexity of the Advisory Committee on Immunization Practices (ACIP) recommendations regarding pneumococcal vaccinations add to the general confusion regarding vaccine administration [5]. ACIP recommends all adults 65 years and older should receive one dose of pneumococcal polysaccharide vaccine (PPSV23) and recommends shared clinical decision-making for use of pneumococcal conjugate vaccine (PCV13) based on risk of exposure to PCV13 serotypes and chronic medical conditions [5].

To improve pneumococcal vaccination in patients ages 65 years and older, providers must become proficient at readily identifying patients who are eligible for vaccination. Yet, quickly identifying patients who are eligible for vaccination in an office visit that typically lasts less than 10 min is hard to do. It is difficult to prioritize multiple recommendations for vaccinating adults, an issue that is magnified by the lack of infrastructure for vaccinating systematically [6,7]. Family physicians routinely care for older adults who present with an increasingly complex problem list laden with chronic conditions. In addition, there are education gaps regarding vaccination schedules among providers and misunderstanding about who assumes primary responsibility for vaccination [8]. Simply knowing that vaccination is warranted is insufficient; knowledge must be accompanied by comprehensive discussion and personalized recommendations. A study identifying barriers and facilitators of pneumococcal vaccination in over 1000 vaccine eligible adults in a variety of settings found that in those unvaccinated for pneumococcal vaccine, three-quarters believed their physician either did not think they should be vaccinated or they did not know their provider’s position on the vaccine [9]. One of the key predictors of vaccination success is the vaccination recommendation from staff in the provider’s office, further attesting to the complexity of influencing the decision to vaccinate [9].

Acknowledging complexity is the first step in solving complex problems. Asking really good questions leads to insight and translating plans into action allows the achievement of desired outcomes [10]. One novel intervention, “academic detailing”, provides a model for working with healthcare practices to solve complex problems. Academic detailing was modeled on pharmaceutical detailing but rather than focusing on sales, focuses on the dissemination of evidence-based information. Academic detailing is an educational outreach by healthcare providers (HCPs) for HCPs to promote best practice. In the current context, academic detailing offers a means for addressing suboptimal adult vaccination rates. A foundational component of academic detailing necessitates baseline knowledge of pneumococcal vaccination schedules and barriers to patient decision making about vaccination recommendations.

The academic detailing model, which dates back to the 1980s, is an evidence-based approach to changing clinical practice, wherein tailored evidence is shared colleague-to-colleague in order to improve practice outcomes [11]. The components of a successful model are: a focused problem, a well-defined target audience, an interactive learning environment, repetition and reinforcement, and the use of brief graphical material [11]. A Cochrane review of academic detailing involving 69 primary studies found that multifaceted detailing had effects that were consistent and clinically relevant [12]. The aim of academic detailing is to affect sustainable practice change, such as building infrastructure for adult vaccinations.

The purpose of this study was to improve pneumococcal vaccination among older adults residing in rural communities with academic detailing using an interprofessional team of physicians, nurses, and pharmacists.

### Theoretical Model

The theoretical model for this study is based upon the Access Framework developed by economists Penchansky and Thomas to better understand barriers to pneumococcal vaccination from the perspective of rural-dwelling older adults [13]. Penchansky and Thomas describe the broad concept of access to healthcare as consisting of five primary components, including: (i) availability, which speaks to volume and supply in the system, (ii) accessibility: defining the ease with which a patient can access service (time, distance, cost to travel), (iii) accommodation, or the willingness of the patient to adapt to the system (e.g., scheduling an appointment, hours of operation, customer service), (iv) affordability, which defines the patient’s ability to pay for services, and (v) acceptability, the likelihood that the patient and provider will feel comfortable working together [13].

Access to healthcare is an increasingly complex American phenomenon, and it is especially complex for older adults. Compared to those who live in urban or suburban communities, older adults in rural communities have a higher prevalence of chronic disease, more disability, fewer healthy behaviors, and a lower life expectancy [14]. In addition, they are more likely to have less access to services, in part due to budget deficits in smaller local governments, struggling small businesses, dwindling economic opportunity, and the migration of young adults to urban settings. Rural older adults are more likely to need resources that they cannot access due to proximity challenges and live in homes that are increasingly unsafe (aging housing stock in need of repair leaves older adults vulnerable to fall risks and mobility challenges).

Given the reality of life in rural America for older adults, the Access Framework helped us to collect information from practices and make recommendations that were designed to improve access to vaccinations. Working with the framework throughout the study sensitized us to the barriers faced by this vulnerable population.

## 2. Materials and Methods

This study utilized a quasi-experimental, single-group, pre-post observational design. Strengthening the Reporting of Observational Studies in Epidemiology (STROBE) guidelines were followed in the design and reporting of this study [15].

### 2.1. Setting and Sample

Two medium sized primary care clinics (two—ten physicians) in two rural counties in Washington served as the setting for this study. Rural was defined using the US Census urban-rural classification system as areas that were not urban (>50,000 people) [16]. Whitman County has a total rural population of 3507 and a rural population density of 10.1 people per square mile [17]. Pend Oreille County has a total rural population of 1104 and a rural population density of 30.9 people per square mile [18]. Sites were recruited through purposive sampling by physician collaborators, who used their extensive knowledge of clinical practice sites throughout the state in their roles as medical school Deans to identify interested sites and make necessary introductions. The sample consisted of all patients aged 65 years or older who were active in the practice, defined as being seen at least once per year. Patients who were younger than 65 years of age and eligible for the pneumococcal vaccination due to comorbidities were excluded from the analysis. Part time providers were excluded from the analysis.

### 2.2. Data Collection

Data were collected from each site for three years prior to the intervention year and for one year following the intervention. These were labelled accordingly as Year 1 (August 2013–2014), Year 2 (August 2014–2015), Year 3 (August 2015–2016), Year 4 (August 2016–2017/Intervention Year), and Year 5 (August 2017–2018). Data were abstracted by contracted information technology professionals from electronic health records and provided in Microsoft Excel spreadsheets.

### 2.3. Intervention

The 12-month intervention began with a needs assessment, from which mutually agreed upon goals were established for the project. These included conducting a workflow assessment of the use of practice-based health information technology (e.g., electronic health record (EHR) and practice management (PM) systems), providing vaccination training to affiliated pharmacy personnel, practice providers and staff, and the creation and distribution of exam-room posters that invited a patient to discuss the need for vaccination with their provider [19,20]. Repeat visits (six in total) were made to each practice at regular intervals to deliver education, discuss needs, select priorities and develop action plans from recommendations. A detailed description of the academic detailing intervention and practice visit schedule has been published elsewhere and summarized in Table 1 [19,20].

The academic detailing intervention was accomplished through multiple visits to the site, each with its own purpose and goals. Multiple education sessions were conducted, as requested, to reacquaint providers with the complex administration schedule. Color printed vaccination schedules were left for providers and staff to post at their workstations to reinforce their learning. In addition, large, laminated exam room posters were widely distributed and posted in exam rooms. The poster gave patients something to read and consider while waiting for the provider. One provider told us that as he walked into an examination room, patients would initiate the conversation by saying, “I was reading about that immunization. Do you think I should have it?” This made it quick and easy for the provider to respond, thereby achieving an important health maintenance goal. Another physician explained.

*“I really enjoyed having those posters. It adds a bit of credibility when I tell my patients about pneumococcal vaccination. What I have found is that if I point to it and advocate for immunization, most of my patients do it.”*

### 2.4. Data Analysis

We hypothesized that vaccination rates would increase over time. Vaccination rates over time were examined by calculating incident proportions for each clinic for each study time period. The eligible clinic population was determined annually, including all participants over the age of 65 that had not received both PPSV23 and PCV13 vaccinations. If there were multiple records of inoculations for an individual, the earliest vaccination date was recorded. Patients receiving the PCV13 vaccination at least one year prior to the PPSV23 vaccination were flagged as having met CDC guidelines, which was labelled as achieving the CDC guideline. A longitudinal descriptive analysis of vaccine rates in the sample population was completed.

To analyze the change in the number of pneumococcal immunizations administered by each individual provider, the list of all providers who administered even one pneumococcal immunization to an adult aged 65 years or older was included.

## 3. Results

**Figure 1:**
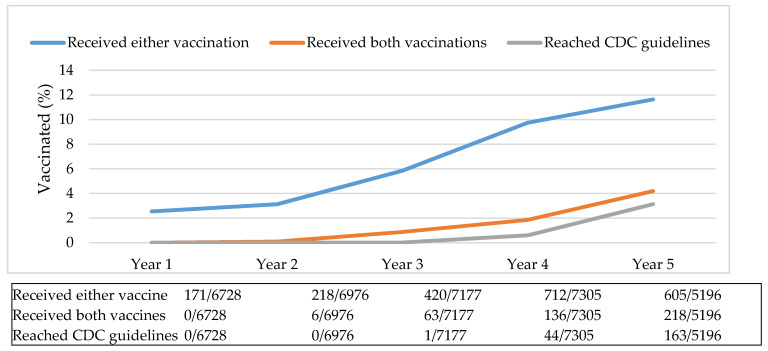
Annual vaccination rates. Abbreviations used: CDC: Centers for Disease Control and Prevention.

The vaccination orders of 22 healthcare providers working in two rural clinics were evaluated. Pneumococcal vaccination rates changed over time. The annual rate of patients vaccinated increased during the five-year study, from 3% in year one to 12% by year five (Figure 1).

**Figure 2:**
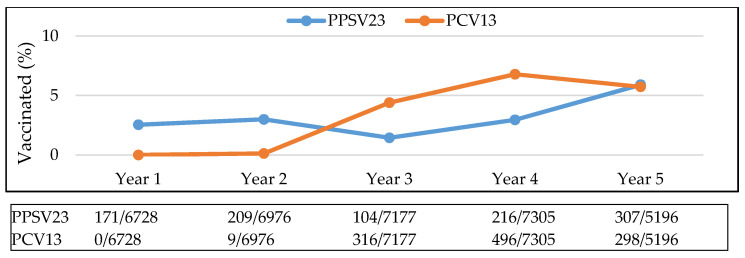
Yearly rate of vaccination. Abbreviations used: PPSV23: pneumococcal polysaccharide vaccine; PCV13: pneumococcal conjugate vaccine.

**Figure 3:**
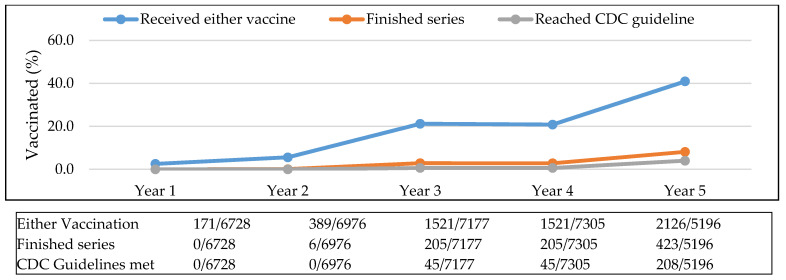
Cumulative proportion of sample population vaccinated. Abbreviations used: CDC: Centers for Disease Control and Prevention.

Annual rates of patients receiving both vaccinations and meeting CDC vaccination guidelines changed slightly over time. The rate at which each vaccine (PPSV23 and PCV13) was given fluctuated over a five-year period, rising sharply as PCV13 became available and then declining after Year 4 (intervention year). The rate of PPSV23 vaccination was lowest in Year 3, before the intervention and increased during Year 4 and thereafter (Figure 2). With the continual increase in annual vaccination rates, the cumulative proportion of the sample population that received vaccination also increased over time (Figure 3).

**Figure 4:**
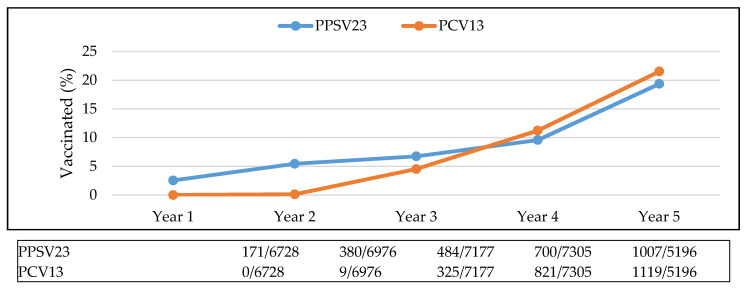
Percentage of population vaccinated, by vaccine. Abbreviations used: PPSV23: pneumococcal polysaccharide vaccine; PCV13: pneumococcal conjugate vaccine.

In Year 1, 3% of the sample population had received either of the vaccinations, whereas by Year 5, 41% were vaccinated. The proportion of the population that received both vaccinations increased from 1% in Year 3 to 8% in Year 5. The proportion of the population that reached the CDC guidelines increased from less than 1% in Year 3 to 4% in Year 5. Ideally, patients received both pneumococcal vaccinations and the percentage of the population that received PPSV23 or PCV13 did increase over the five-year period (Figure 4).

Two and a half percent of the population had the PPSV23 vaccination in Year 1, increasing to 19.4% by Year 5. Less than one percent of the population had the PCV13 vaccine in Year 2, increasing to 21.5% by Year 5.

There were noted differences in the number of vaccinations given by providers over time. All providers who administered even one pneumococcal immunization to an older adult were included in the analysis. There was variation within providers: average vaccination rate per year ranged from 0.5 to 61 (Table 2); and between providers: whereby providers continued to give more vaccinations per year with Year 1 averaging 13.9 vaccines per provider to Year 5 averaging 31.6 vaccines.

## 4. Discussion

As expected, an academic detailing intervention designed to focus attention on pneumococcal vaccination served to increase the number of PPSV23 and PCV13 vaccines administered during the year-long intervention. An increase in PPSV23 was noted in the 12 months following the intervention.

**Figure 5:**
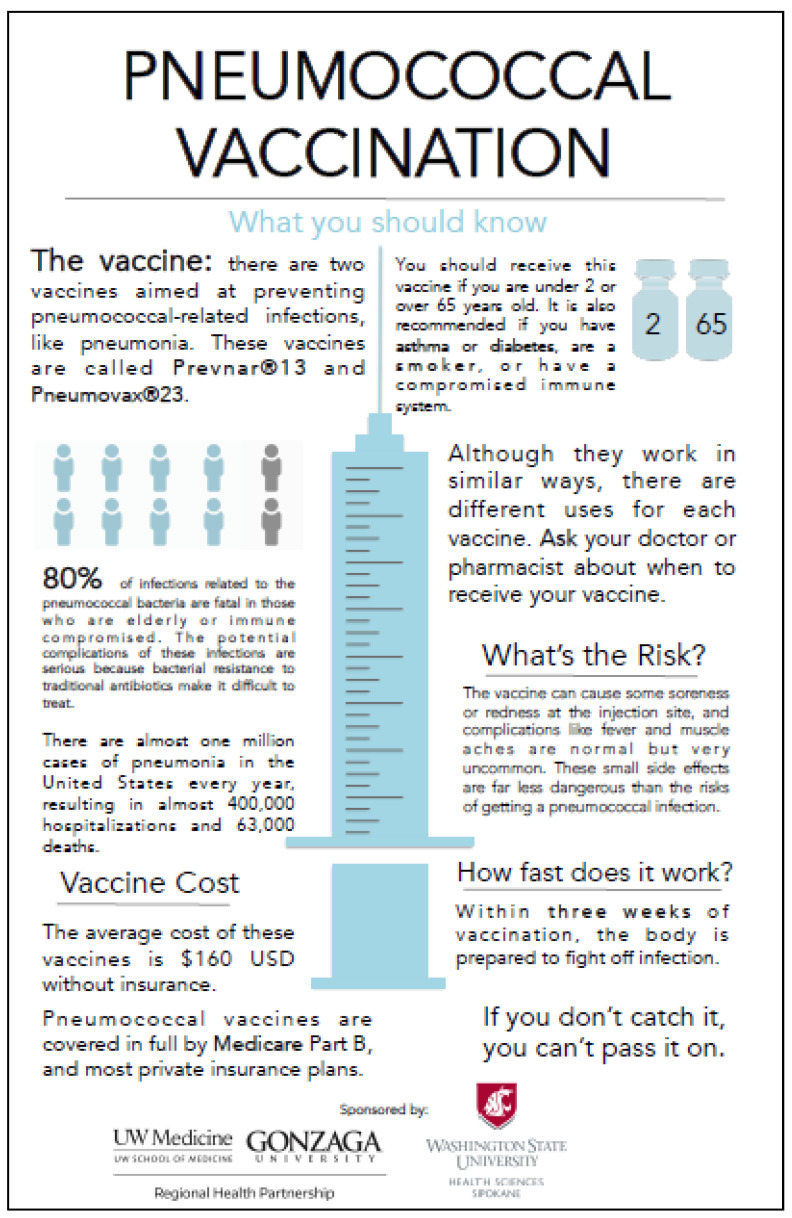
The examination room poster.

As the older adults who were vaccinated in this study managed to attend a family practice visit (accessibility, accommodation) and pay for the visit and vaccine (affordability), we assume the rise in vaccination rates was due largely to the offer to vaccinate (availability) and the willingness of the provider and the patient to engage in meaningful dialogue about it (acceptability). The academic detailing intervention used in this study may have increased provider and staff knowledge of the complex pneumococcal vaccination schedule thereby increasing available opportunities for vaccination. In fact, the exam room posters (Figure 5) were the element in the academic detailing intervention that clinic providers and staff expressed most satisfaction with [19,20].

However, vaccine uptake is not seen as limited to patients. HCP attitude toward vaccination may greatly contribute to overall uptake. We know, for example, that HCP willingness to take seasonal influenza vaccinations are influenced by mandates and that HCP willingness to take, and recommend, the COVID-19 vaccination is influenced by professional role and amount of education [21,22]. This study was designed to examine access from the patient perspective, so limited data was collected on the HCP. Future studies should include an evaluation of “acceptability” from the HCP perspective, as it is reasonable to assume that the HCP position on vaccination greatly influences their patients’ perspective.

In an attempt to make vaccination more accessible to patients, clinic workflows were re-organized so that medical assistants who roomed patients were altered (by a flag in one clinic and a report in the second clinic), to the need for vaccination. One clinic took this idea a step further and sent notes to patients from the EHR report notifying them that they were eligible to receive this highly recommended vaccination and encouraged them to make an appointment to discuss it. These enhanced workflows provided an opportunity to introduce the idea before the provider saw the patient and communicated a sense of importance and regularity to pneumococcal vaccination, increasing the likelihood the patient would be comfortable receiving it. Exam room posters and EHR alerts were seen as a way to promote acceptability of pneumococcal vaccination. Subsequent studies are needed to better understand this phenomenon; does pre-encounter messaging promote patient compliance or informed dialogue?

The results of this work demonstrated an increase in the average number of pneumococcal immunizations given by clinic providers from 13.9 vaccines per provider in Year 1 to 31.6 vaccines in Year 5. However, there is variation among providers whereby the mean number of vaccinations ranges from a high of 61 per year to a low of one. Any provider who administered even one pneumococcal immunization to an adult aged 65 years or older was included in the study, which may have unduly highlighted a difference that relates to something other than the proper administration of evidence-based guidelines for the care of older adults. Future work should further sub-divide provider and patient type to more fully examine these patterns.

Despite widespread national vaccination efforts, national pneumococcal vaccination rates are still below the Healthy People 2020 goal of 90% for adults ages 65 years and older [4]. According to Healthy People 2020, the percentage of U.S. adults aged 65 years and older who received at least one pneumococcal immunization increased from 59.7% in 2013 to 69% in 2017 [4]. According to a national phone survey conducted by the CDC in the State of Washington, the percentage of adults aged 65 and older who received at least one pneumococcal immunization rose from 73.4% in 2013 to 79.4% in 2017 [23]. The statewide increase of 8% is much lower than the change noted in this study, where vaccination rates moved from 3% to 41% during the same time period. Although the percentage of adults vaccinated was much lower in the study population, the increase over the project period of (1266%) was much higher than the change in the U.S. rate (15.6%) and Washington State (8%) rate. Further research is needed to determine what the difference among these rates could be attributed to.

Successfully increasing vaccination rates in rural communities will require enthusiastic involvement from the broader healthcare team. Improving access to vaccines may provide a path to closing the gap between rural and urban healthcare. Pharmacists routinely administer vaccinations in Washington state within their scope of practice and are often able to prescribe vaccinations through pre-negotiated collaborative drug therapy agreements (RCW 18.64.011 [11]) [24]. In rural communities where people are likely to know each other well, some community pharmacists reported being reluctant to “get into the immunization scene” because they feared “taking business away” from someone they knew well (and were sometimes related to or had a financial interest in working well with). Yet, encouraging pharmacy-administered vaccination in rural communities makes sense when considered through the lens of the Access Framework. Offering access to evening or weekend hours in places that allow walk-in visits without appointments at the community pharmacy or grocery store may better suit some older adults’ preferences and schedules.

Finally, the academic detailing intervention provided an innovative way for academicians and clinicians to work together to achieve an important patient-centered goal. This study relied on multidisciplinary partnerships which was helpful in securing buy-in from different clinic personnel. The multi-modality nature of the intervention helped target different needs of providers, office staff, and patients. In addition, the interprofessional project served to strengthen partnerships and relationships among our community’s health-sciences programs.

### Limitations

There are methodological limitations to this study. First, there is no causation associated with the rise in vaccination rates during the intervention year due to the nature of the design. Other types of designs may have allowed for a comparison or control group. A proposal for future work has been written which would allow for a control group (another practice) and the identification of specific aspects of academic detailing associated with higher rates of vaccination (e.g., poster, training, 1:1 coaching) through a staged randomized controlled design. Second, there is a possibility that the Hawthorne effect, whereby the observation of researchers modifies participants’ behavior, is responsible for some of the observed effect. Third, the year one denominator did not account for the patients who may have been vaccinated the year prior, so reported first year rates could be artificially low. Fourth, this study did not account for other things that may have been responsible for enhanced patient or provider awareness or knowledge (e.g., continuing medical education events or television commercials), thereby increasing vaccination rates independent of the study. Fifth, demographic information for patients and providers was not collected by the study team. Although both clinics were in rural and underserved areas where the majority of the population is Caucasian, having information about patient gender, age (other than being over 65), education, and socioeconomic data could provide additional insight.

## 5. Conclusions

An interprofessional academic detailing intervention was well accepted and increased pneumococcal vaccination rates among rural-dwelling older adults. Academic detailing provides an empirically based flexible way to meet the needs of practicing clinicians. It can be used to address gaps in knowledge or infrastructure that are frequently cited as barriers to adult vaccination. Education alone has proven ineffective at changing behavior and improving vaccination rates. Given the alarming disparities in health outcomes for rural dwelling older adults, partnerships such as between academic centers and rural healthcare practices are desperately needed to improve healthcare access and outcomes.

## Figures and Tables

**Table 1:** Schedule of academic detailing visits.

Visit Number	Visit Purpose
1	Met with physician leaders to enroll practices; conduct needs assessment to determine what would be helpful
2	Shadowed practice providers, nurses, and medical assistants to observe immunization workflow and documentation
3	Met with clinical physicians, nurses, pharmacists, administrators to conduct 30-min presentation about pneumococcal immunization use; delivered exam room posters
4	Met with practice physicians, nurses, administrators to re-visit needs assessment results and determine if additional assistance would be helpful
5	Met with practice leaders to discuss best practices for using EHR/PM, resulting from workflow analysis
6	Met with practice medical assistants to provide additional training on pneumococcal immunization schedules

Abbreviations used: EHR: electronic health record; PM: practice management.

**Table 2:** Number of vaccinations administered organized by provider.

Provider	Vaccine	Number of Vaccines Administered per Year					Total	Total Vaccines Combined	Mean
		Year 1	Year 2	Year 3	Year 4	Year 5			
Provider A	PCV13	0	0	52	224	131	407	610	61
	PPSV23	54	32	5	21	91	203		
Provider B	PCV13	0	3	84	72	19	178	359	35.9
	PPSV23	18	68	21	37	37	181		
Provider C	PCV13	0	2	44	41	27	114	333	33.3
	PPSV23	37	35	16	81	50	219		
Provider D	PCV13	0	3	40	66	33	142	259	25.9
	PPSV23	21	26	11	12	47	117		
Provider E	PCV13	0	0	3	10	5	18	178	17.8
	PPSV23	37	29	19	22	53	160		
Provider F	PCV13	0	2	0	25	19	46	157	15.7
	PPSV23	13	22	13	36	27	111		
Provider G	PCV13	0	18	3	4	2	27	138	13.8
	PPSV23	33	15	7	26	30	111		
Provider H	PCV13	0	16	1	1	0	18	113	11.3
	PPSV23	30	13	5	21	26	95		
Provider I	PCV13	0	0	10	6	12	28	65	6.5
	PPSV23	5	5	2	11	14	37		
Provider J	PCV13	0	0	9	9	5	23	52	5.2
	PPSV23	4	4	3	7	11	29		
Provider K	PCV13	0	1	0	0	0	1	51	5.1
	PPSV23	18	9	6	6	11	50		
Provider L	PCV13	0	4	5	6	0	15	48	4.8
	PPSV23	15	5	5	7	1	33		
Provider M	PCV13	0	0	2	2	14	18	28	4.7
	PPSV23	0	0	1	1	8	10		
Provider N	PCV13	0	0	0	3	4	7	22	2.2
	PPSV23	4	4	3	3	1	15		
Provider O	PCV13	0	0	0	6	0	6	13	1.625
	PPSV23	1	2	2	2	0	7		
Provider P	PCV13	0	0	0	0	9	9	13	2.2
	PPSV23	1	1	0	0	2	4		
Provider Q	PCV13	0	0	3	4	0	7	13	1.3
	PPSV23	2	1	1	1	1	6		
Provider R	PCV13	0	0	0	0	3	3	11	1.1
	PPSV23	2	2	2	1	1	8		
Provider R	PCV13	0	0	0	0	0	0	7	1.2
	PPSV23	3	0	0	3	1	7		
Provider T	PCV13	0	0	0	0	0	0	7	3.5
	PPSV23	7	0	0	0	0	7		
Provider U	PCV13	0	0	0	0	0	0	2	0.5
	PPSV23	1	0	1	0	0	2		
Provider V	PCV13	0	0	0	0	1	1	1	0.5
	PPSV23	0	0	0	0	0	0		
Total		306	322	379	777	696	2480		
Mean		13.9	14.6	17.2	35.3	31.6			

## Data Availability

The data presented in this study are available on request from the corresponding author.
